# Patients’ experiences with intensive combination treatment strategies for early rheumatoid arthritis: a longitudinal qualitative study embedded in the carera trial

**DOI:** 10.1186/1472-6963-14-S2-P146

**Published:** 2014-07-07

**Authors:** Sabrina Meyfroidt, Kristien Van der Elst, Diederik De Cock, Johan Joly, René Westhovens, Marlies Hulscher, Patrick Verschueren

**Affiliations:** 1 Department of Development and Regeneration, KU Leuven, Leuven, Belgium; 2 Department of Public Health and Primary Care, KU Leuven, Leuven, Belgium; 3 Rheumatology, University Hospitals Leuven, Leuven, Belgium; 4 Scientific Institute for Quality of Healthcare, Radboud University Medical Center, Nijmegen, The Netherlands

## Background

The current recommendations for early Rheumatoid Arthritis (eRA) management focus on achieving clinical remission as soon as possible with an early and intensive treatment. Understanding patients’ experiences and ideas regarding their treatment could make healthcare professionals (HPs) more aware of and timely responsive to patients’ preferences, which might result in a better treatment adherence, an improved health status and higher satisfaction with care.

## Objectives

To gain a longitudinal understanding of the experiences of patients with eRA with intensive combination treatment strategies (ICTS) at two time points in the early phase of the treatment process.

## Materials and methods

We performed a longitudinal, qualitative study embedded in the CareRA (Care in eRA) trial, a multicentre RCT comparing different combinations of conventional DMARDs plus step-down bridging schemes of glucocorticoids for eRA. Patients with eRA participating in the CareRA trial were purposively sampled. At time point 1 (TP1), 4-6 months after initiation of ICTS, 26 patients were interviewed individually. At time point 2 (TP2), at least one year after treatment initiation, 14 patients of the same study sample participated in 1 out of 3 focus groups. Each interview was audio-recorded, literally transcribed and thematically coded using the constant comparative method.

## Results

Four main themes were observed regarding patients’ experiences with ICTS. Firstly, patients expressed pre-occupations and feelings about ICTS that changed between both time points, such as fear of side effects at TP1 that diminished at TP2. Secondly, the need for additional information differed among individual patients and shifted from TP1 to TP2. The sources of information most commonly used over time were HPs, relatives and the internet. Thirdly, patients reported about their relationship with HPs and the need to trust them to follow their advice, especially at TP1. Lastly, patients described their self-management strategies and how quickly ICTS was integrated into their daily routine.

## Conclusions

Patients’ experiences with ICTS changed as they progressed through the early phase of the treatment process. Despite concerns at treatment initiation, most patients expressed positive experiences with ICTS. These study findings could facilitate the application of ICTS in daily clinical practice for eRA.

